# Gut health immunomodulatory and anti-inflammatory functions of gut enzyme digested high protein micro-nutrient dietary supplement-Enprocal

**DOI:** 10.1186/1471-2172-10-7

**Published:** 2009-01-31

**Authors:** Jagat R Kanwar, Rupinder K Kanwar

**Affiliations:** 1 BioDeakin, Institute for Technology & Research Innovation (ITRI), Deakin University, Geelong Technology Precinct (GTP), Pigdons Road, Waurn Ponds, Geelong, Victoria 3217, Australia

## Abstract

**Background:**

Enprocal is a high-protein micro-nutrient rich formulated supplementary food designed to meet the nutritional needs of the frail elderly and be delivered to them in every day foods. We studied the potential of Enprocal to improve gut and immune health using simple and robust bioassays for gut cell proliferation, intestinal integrity/permeability, immunomodulatory, anti-inflammatory and anti-oxidative activities. Effects of Enprocal were compared with whey protein concentrate 80 (WPC), heat treated skim milk powder, and other commercially available milk derived products.

**Results:**

Enprocal (undigested) and digested (Enprocal D) selectively enhanced cell proliferation in normal human intestinal epithelial cells (FHs74-Int) and showed no cytotoxicity. In a dose dependent manner Enprocal induced cell death in Caco-2 cells (human colon adencarcinoma epithelial cells). Digested Enprocal (Enprocal D: gut enzyme cocktail treated) maintained the intestinal integrity in transepithelial resistance (TEER) assay, increased the permeability of horseradish peroxidase (HRP) and did not induce oxidative stress to the gut epithelial cells. Enprocal D upregulated the surface expression of co-stimulatory (CD40, CD86, CD80), MHC I and MHC II molecules on PMA differentiated THP-1 macrophages in coculture transwell model, and inhibited the monocyte/lymphocyte (THP-1/Jurkat E6-1 cells)-epithelial cell adhesion. In cytokine secretion analyses, Enprocal D down-regulated the secretion of proinflammatory cytokines (IL-1β and TNF-α) and up-regulated IFN-γ, IL-2 and IL-10.

**Conclusion:**

Our results indicate that Enprocal creates neither oxidative injury nor cytotoxicity, stimulates normal gut cell proliferation, up regulates immune cell activation markers and may aid in the production of antibodies. Furthermore, through downregulation of proinflammatory cytokines, Enprocal appears to be beneficial in reducing the effects of chronic gut inflammatory diseases such as inflammatory bowel disease (IBD). Stimulation of normal human fetal intestinal cell proliferation without cell cytotoxicity indicates it may also be given as infant food particularly for premature babies.

## Background

The ability to eat sufficient amounts orally is inversely associated with the extent of frailty and studies have indicated that nutritional intake is often compromised in the elderly [1]. Poor gut health and secondary health issues as a consequence are matters of nutritional attention for the elderly, infants and immune compromised persons. Diet is a factor that can be extremely varied in terms of quantity, quality, frequency and duration of meals in individuals, but that can also be modified with targeted dietary interventions to produce the best results for any individual [2]. Diets of the elderly are generally low in protein, low in energy and deficient in a range of micro-nutrients [1,3,4]. High protein diets, in particular those based on whey proteins, are being marketed to improve the health status of the elderly [4] as well as other target groups e.g. athletes and babies. Furthermore micro-nutrients including anti-oxidants e.g. Vitamins A, C and zinc are also being used for fortification or as dietary supplements for healthy older patients [5]. There are growing number of compositional foods under development, being patented or emerging in the market that target specific nutritional deficiencies beyond the traditional infant formula market. Recent examples include WO2007/070754 a high protein dietary supplement for treating various diseases associated with protein deficiency (Novalife); and WO2007/022991 compositions for enhancing vascular integrity, cellular survival and reducing apoptosis in ischema (Nestec).

Enprocal, a recently formulated supplementary food is designed to meet the nutritional needs of the frail elderly and to be delivered to them in common every day foods. Enprocal's formulation was developed by specialised aged care dieticians and a food scientist experienced in powder based food supplements. The ingredients of Enprocal such as dairy-based proteins (whey protein concentrate, skim milk powder, whole milk powder), vitamins and minerals (including calcium, zinc and vitamins C, D, B group and A), vegetable oils and inulin (fibre) were selected to meet the nutritional profile of elderly specified though dietetic input (Table 1). With whey protein concentrate (WPC) (80% protein) as its key ingredient it provides i) the highest-quality protein (as measured by protein efficiency ratio (PER), ii) rapid protein digestion, iii) essential amino acids, in particular cysteine, threonine and leucine that may be required in increased levels in the elderly [6,7], iv) cysteine to improve glutathione status and muscle protein synthesis, v) high concentrations of branched chain amino acids, and vi) minor bioactive components such as immuno-globulins, growth factors, and anti-microbial proteins and peptides to assist in effective gut function [6,8-12]. Today, whey is a popular dietary protein supplement purported to provide antimicrobial activity, immune modulation, improved muscle strength and body composition, and to prevent cardiovascular disease and osteoporosis [reviewed in [8,11,12]]. Enprocal with WPC as its key ingredient therefore contains high levels of constituent proteins including beta-lactoglobulin, alpha-lactalbumin, lactoferrin, lactoperoxidase and glycomacropeptide which have been demonstrated a range of immune-enhancing properties [11,12]. In addition, whey has the ability to act as an antioxidant, antihypertensive, antitumor, hypolipidemic, antiviral, antibacterial, and chelating agent [6,8,11,12]. Whey proteins have been reported to enhance digestion and gut function [8,11-13] as well as glutathione production and immune function, hence increasing dietary availability may promote general health in a variety of ways [14,15].

**Table 1:** Composition of Enprocal.

**Ingredients**	**Composition per 100 g**
Whey protein concentrate	41.4 g

Vegetable fibre (inulin)	10.2 g

Vegetable oil	8.2 g

Vitamin A	548 μg

Thiamin (Vitamin B1)	0.8 mg

Riboflavin (Vitamin B2)	1.9 mg

Folate	173 μg

Vitamin B6	1.0 mg

Vitamin B12	2.9 μg

Vitamin C	57 mg

Vitamin D	9 μg

Vitamin E	4.2 mg

Calcium	1142 mg

Iron	3.9 mg

Magnesium	232 mg

Phosphorus	780 mg

Zinc	10.7 mg

Developing and maintaining a healthy intestinal tract is a pre-requisite for general health and aiding disease prevention. The gut epithelial cells lining of the intestinal lumen are the first point of contact between intestinal contents and the rest of the body. It is at this interface that nutrition, environment and genetics come together to determine gut health. The intestinal mucosa is lined by a monolayer of intestinal epithelial cells joined together at their apical poles by tight junctions, and supported by an extensive and complex immune system and transcellular pathways for nutrient absorption [16]. The mucosal surface of the gastrointestinal tract forms a barrier that separates the luminal contents from the effector immune cells underneath. With the emergence of the concept of immunophysiology, it is clear that the function of the intestinal epithelium is affected by many immune cell types [17]. Any abnormal activation of such immune cells (macrophages) overproduces the inflammatory cytokines e.g., tumor necrosis factor (TNF)-α, interleukin (IL)-1β and IL-6 that cause destructive cell damage to the intestinal epithelial monolayers and subsequent mucosal inflammation leading to inflammatory bowel disease (IBD) such as Crohn's disease (CD) and ulcerative colitis (UC) [18,19]. Cellular injury from reactive oxygen species (ROS) is implicated in a wide variety of gut diseases and pathologic conditions [20]. There is a growing body of evidence that oxidative stress may contribute to gut inflammation, ulcers, inflammatory bowel disease, and leaky gut syndrome. Newborn and the elderly are more prone to oxidant injury [20-22].

The present study was aimed to assess the potential of Enprocal to improve gut and immune health functions. We investigated specifically whether Enprocal, after digestion with a cocktail of gut enzymes maintained gut cell proliferation and mucosal integrity, possessed immunomodulatory (monocyte/macrophage and T cell activation and cytokine production), anti-inflammatory (immune-gut cell adhesion) and anti-oxidative properties. We sought to achieve conditions as close as possible to those of the human digestive tract by employing *in vitro*, most widely used human gut epithelial cells alone (Caco-2 and FHS Int 74) or in coculture with human immune THP-1 (monocytes) and Jurkat (T lymphocytes) cells in transwell system.

FHs 74 Int and Caco-2 cell lines used worldwide by the researchers were employed as the models of human intestinal epithelial cells in the study. FHs 74-Int cells, used in the study were although derived from normal human fetal intestine but have been reported to show mature epithelial-like characteristics [23]. The growth-promoting activities of EGF, TGF-α IGF-I and insulin were examined using the same intestinal cells [24]. Caco-2 cells (derived from human colon adenocarcinoma) have gained enormous popularity as a reliable and high-throughput *in vitro *model system for the study of human intestinal permeability and evaluation of a large number of drug candidates for their intestinal absorption [25,26]. For orally administered compounds, permeability through Caco-2 cell monolayers correlates well with human *in vivo *absorption [26-28]. Caco-2 monolayers can display electrical properties typical of either small intestinal or colonic enterocytes [27]. Caco-2 cells are able to differentiate spontaneously in culture and display a small intestine-like (villus) phenotype. The differentiated cells polarise, form microvilli and secrete enzymes associated with the enterocyte brush border such as sucrase-isomaltase, alkaline phosphatase, alkaline phosphatase, lactase and aminopeptidase [29]. This cell line thus represents an appropriate model for the study of transport mechanisms related to the intestinal barrier and for investigating diverse problems of nutrients bioavailability and absorption without concern for differences between human and animals [30].

Our study, provides the first report on the bioactivity of digested Enprocal components which may be released through enzymatic action from its key ingredients such as whey protein concentrate for gut epithelial cell growth, integrity, absorptive, immunomodulatory, anti-inflammatory and anti-oxidative properties.

## Results

### Digested Enprocal (Enprocal D) maintains cell growth, enhances cell proliferation and shows no cytotoxicity in normal human intestinal epithelial cells

**Figure 1:**
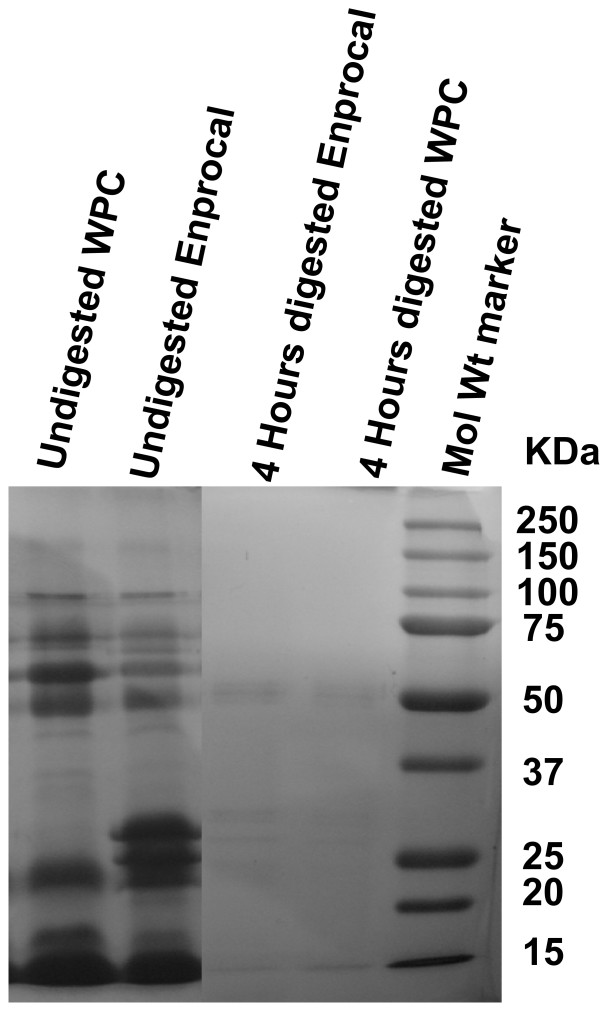
**SDS-PAGE analysis of Enprocal and whey protein concentrate (WPC) digested with gut enzyme cocktail (pH7.2) for 4 hours**.

**Figure 2:**
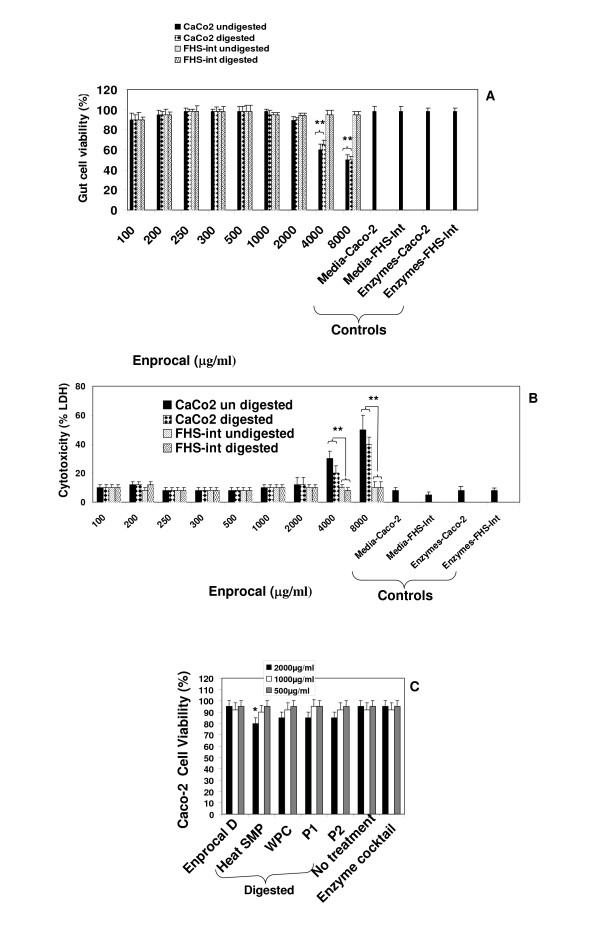
**Cell viability (A), Cell cytotoxicity (B) of Caco-2 and normal human FHs74-Int cells treated with undigested and digested Enprocal (Enprocal D) 100–8000 μg/ml of each for 48 hours**. Cell viability was determined by trypan blue exclusion assay. Cell cytotoxicity was determined by LDH release assay. (C) Cell viability (trypan blue exclusion assay) of Caco-2 monolayers treated with Enprocal D (500–2000 μg/ml) and with other digested milk product controls. All treatments were performed in triplicate and assay was repeated three times independently with similar results. The mean for representative experiment was calculated and presented as a mean ± SD values. ** Indicates a highly significant P < 0.001 value from the control with media only. *Indicates a significant P < 0.05 value from the control with media only.

**Figure 3:**
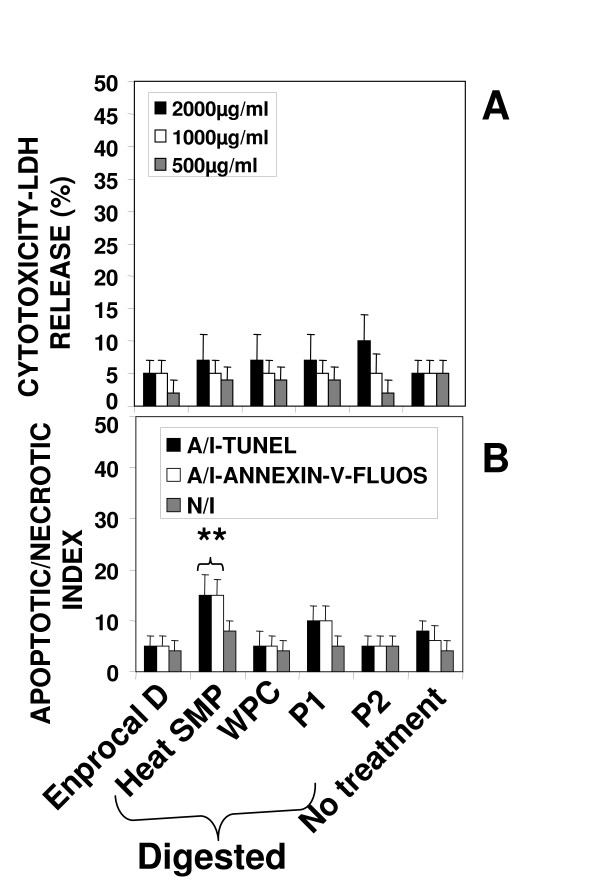
**Cell cytotoxicity determined by LDH release assay (A) and cell death (apoptosis/necrosis) (B) following treatment with 500–2000 μg/ml of Enprocal D and other digested milk product controls**. Cells were treated for 48 hours with milk products and stained by TUNEL analysis for apoptotic cells, Annexin-V-fluos analysis and counterstained with propidium iodide to reveal necrotic cells. Cell death is shown here in terms of apoptotic (A/I) and necrotic indices (N/I). All treatments were performed in triplicate and assay was repeated three times independently with similar results. The mean for representative experiment was calculated and presented as a mean ± SD values. ** Indicates a highly significant P < 0.001 value from the control with media only. *Indicates a significant P < 0.05 value from the control with media only.

**Figure 4:**
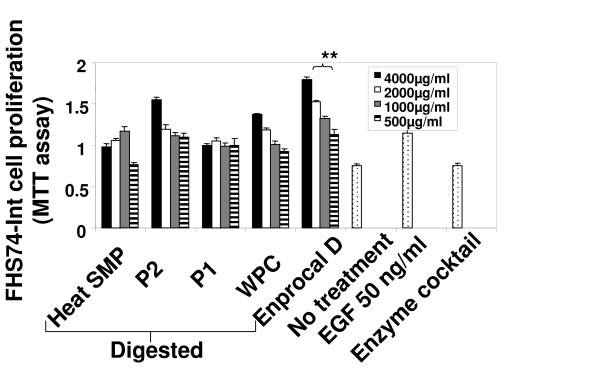
**Effect of Enprocal D and other digested milk product controls on cell proliferation of normal human intestinal cells (FHs74-Int) determined by MTT assay**. All treatments were performed in triplicate and assay was repeated three times independently with similar results. Each bar presented in the histogram was mean ± SD values of all experiments in triplicates. **P < 0.001 is the highly significant value from the control with media only or enzyme cocktail. *P < 0.05 is the significance value from the control with media only or enzyme cocktail.

Near complete digestion of Enprocal and other digested milk product controls was achieved after 4 hours incubation with gut enzyme cocktail mixture (Fig 1). In our preliminary experiments we treated Enprocal with the gut enzyme cocktail at different enzyme cocktail: protein ratios at pH 7.0–7.4. A 1:50 ratio of enzyme cocktail: protein at pH 7.2 was found to be most optimal and complete digestion was achieved after 6 hours. Further it was deemed necessary to find a concentration range which would not kill/disrupt the cell monolayers needed to test in bioassays on permeability, immunomodulation, cell adhesion, and oxidative stress. We treated Caco-2 and FHs 74 Int cells with digested and undigested samples of Enprocal at different concentrations (100–8000 μg/ml) to determine the effect on cell viability and growth determined by trypan blue exclusion assay (Fig 2A). With both Enprocal undigested and digested samples, the Caco-2 cell viability as compared to that of FHs 74 Int was significantly reduced in (P < 0.001) at concentrations 4000 and 8000 μg/ml. There was visible reduction in Caco-2 viability with Enprocal D compared to undigested Enprocal. However, these levels did not reach any significance. There was no effect of all concentrations tested in the range of 100–8000 μg/ml on the cell viability of normal intestinal FHs 74 Int cells. This was further confirmed in the LDH released cytotoxicity assay (Fig 2B). There was no significant increase (P > 0.05) in the cytotoxicity as compared to control (media only) when FHs 74 Int cells were treated with digested and undigested samples of Enprocal from 100–8000 μg/ml. The enzyme cocktail showed no effect of its own on cell viability and cytotoxicity in both the cell lines. Although there was no significant difference between the cell viability and cytotoxicity effects of digested and undigested Enprocal samples but to mimic the *in vivo *system we selected the digested form for bioassays. From the cell viability and cell cytotoxicity results 500–2000 μg/ml was selected as the optimal concentration range for the bioassays. The other control milk product digests at these concentrations were tested for their effects on cell viability in trypan blue assay (Fig 2C). When compared to media only control (no treatment) and Enprocal D, heat SMP digest samples reduced significantly (P < 0.001) the Caco-2 cell viability at 2000 μg/ml. The control milk products were further tested in LDH release assay (Fig 3A), and cytotoxicity levels were below 10% and not significant (P > 0.05) at 2000 μg/ml concentration when compared with Enprocal D and media only control. In TUNEL and Annexin-V-fluos assays, Enprocal D did not significantly (P > 0.05) increase the apoptotic index (AI) and necrotic index (NI), however heat SMP induced significant (P < 0.01) AI as compared to media only control in Caco-2 cells (Fig 3B). In a dose dependent manner (500–4000 μg/ml) Enprocal D increased significantly (P < 0.001) the proliferation of FHs 74 Int cells (Figure 4), determined by MTT assay, as compared to untreated and positive control (EGF 50 ng/ml) cells. When compared with other control products this effect was significant (p < 0.001) except for P2 (500 μg/ml) where the difference was insignificant between Enprocal and P2. However the cell proliferation of Caco-2 cells was decreased significantly following treatment with Enprocal D (data not shown).

### Enprocal D maintains the mucosal integrity, increases the expression of ZO-2 (tight junction protein) and macromolecular absorption

**Figure 5:**
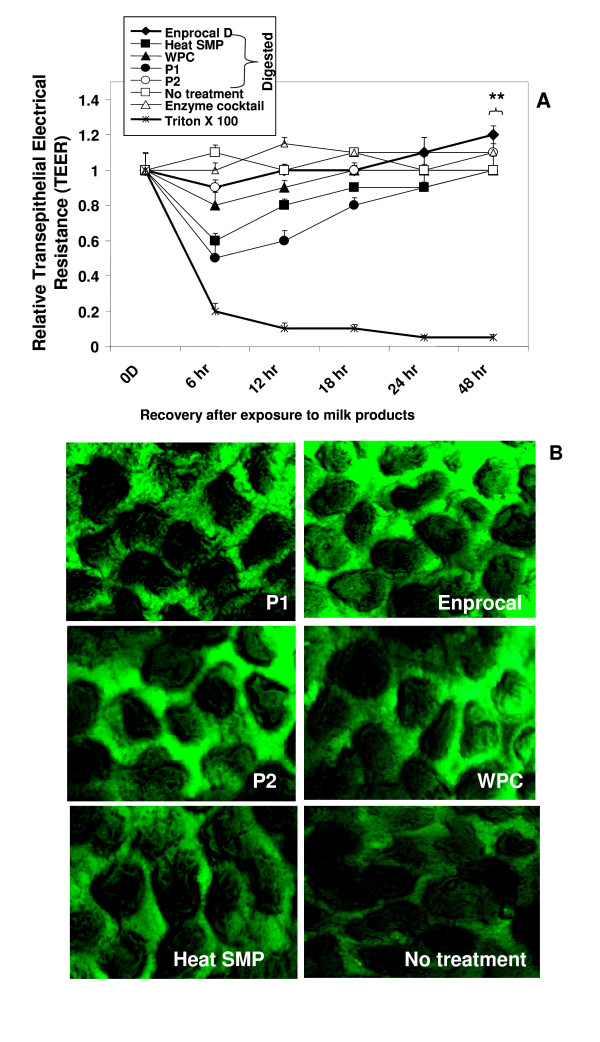
**Effect of Enprocal D and other digested milk product controls on transepithelial electrical resistance (TEER) (A), tight junction protein-ZO-2 expression (immunofluorescence in confocal microscopy) (B)**. Caco-2 monolayers (21 day old, differentiated cells) were incubated with 500 μg/ml of test samples and control (no treatment) for 48 hours as indicated. All treatments were performed in triplicate and assay was repeated three times independently with similar results. The effect on epithelial barrier integrity was measured (TEER results in ohms-cm^2^) and mean ± SD to relative TEER values were calculated with respect to media only controls and plotted. Significant differences (*P, < 0.05) and highly significant differences (**P, < 0.001) between the relative TEER levels as compared to control cells are indicated by asterisks.

Intestinal Caco-2 monolayers grown on Millipore cell culture inserts in transwell system were used to measure the transepithelial resistance (TEER) following treatments with Enprocal D and other digested milk product controls at a concentration of 500 μg/ml (Fig 5A). At 0 hour before treatment, the Caco-2 monolayers after 21 days of growth were found to be fully differentiated to enterocytes and polarized, continuous without any gaps and displayed TEER values 520 ± 20 ohm.cm^2 ^in control growth media. Figure 5A showed the TEER values of cell monolayers following different treatments relative to control (media only). The most significant (P < 0.01) decrease was observed in all treatments after the first 6 hours except for Enprocal D and P2 (P > 0.05). Both P2 and Enprocal D were able to recover the TEER values at 12 hours and the increase was maintained further till 48 hours duration (end of experiment) as compared to other control milk products. However, TEER resistance values for Enprocal D were found to increase further after 24 hours. P1 was found to decrease significantly (P < 0.001) the TEER after 24 hours. The gut enzyme cocktail had no effect on the TEER when compared with media only control. The Triton-X, known to disrupt the cell monolayer, was used as the positive control. It reduced significantly (P < 0.001) the TEER resistance at 6 hours which were not recovered throughout the experimental period. The monolayer integrity results were further confirmed by expression of tight junction protein -ZO-2. Figure 5B shows an upregulation of ZO-2 expression on Caco-2 monolayers treated with 500 μg/ml of Enprocal and P2 digests for 12 hours.

In Horseradish Peroxidase (HRP) flux assay, Enprocal D aided macromolecular transport through the Caco-2 cell monolayer (Table 2). HRP flow through increased with time after treatment with Enprocal D and other milk product digests. After 4 hours significant (P < 0.05) HRP flux increase (1.5 fold) as compared to media only control was observed in Enprocal treated cells which increased further to 2 and 3.9 fold after 8 and 16 hours respectively. Among other products 1.5, 1.8, 1.3 and 2.1 fold increase was observed for heat SMP, WPC, P1 and P2 respectively at 8 hours while significant (P < 0.001) increase, 2, 2.4, 2.6 and 4.4 fold occurred at 16 hours for heat SMP, WPC, P1 and P2 respectively. As expected the positive control Triton X, showed 9.7 fold increases at 4 hours which remained consistent till 16 hours. The enzyme cocktail mixture had no effect when compared with untreated cells.

**Table 2:** Effect of Enprocal D and other digested milk product controls on macromolecular absorption determined by Horseradish Peroxidase (HRP) flux assay.

**Ingredients**	**4 hr (Mean ± SD)**	**8 hr (Mean ± SD)**	**16 hr (Mean ± SD)**
Enprocal	12.5 ± 1.8*	17.5 ± 1.3*	35.5 ± 1.6**

WPC	7.9 ± 0.74	15.6 ± 0.7*	22.2 ± 0.7**

P1	6.5 ± 0.8	10.9 ± 0.6	24.5 ± 0.7**

P2	10.5 ± 1.4	18.5 ± 1.2*	40.4 ± 1.3**

Heat SMP	8.5 ± 0.6	12.4 ± 0.5	18.4 ± 0.5**

No treatment	8.3 ± 1.3	8.5 ± 1.1	9.1 ± 2.6

Enzyme cocktail	8.5 ± 1.2	8.5 ± 1.2	8.7 ± 1.3

Triton-X 100	80.5 ± 2.8**	85.3 ± 2.5**	88.5 ± 2.6**

HRP flux results are shown in percentage for 4–16 hours determined in the basolateral compartment. All samples were assayed in three independent assays each performed in triplicate and the mean for all experiments was calculated and presented as a mean ± SD values.

** Indicates a highly significant P < 0.001 value from the control with media only (no treatment).

*Indicates a significant P < 0.05 value from the control with media only (no treatment). Triton-X 100 is a known cell disruptor and act as a positive control in HRP flux assay

### Anti-oxidant activities of Enprocal D

**Figure 6:**
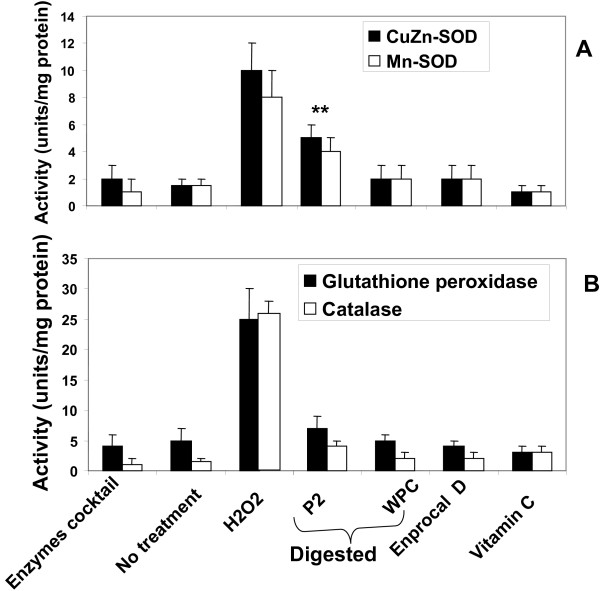
**Antioxidant enzymes activities for copper zinc superoxide dismutase (CuZnSOD) and manganese superoxide dismutase (MnSOD) (A), glutathione peroxidase (GPx), and catalase (CAT) (B) were determined after treating Caco-2 cells with 500 μg/ml of Enprocal D and compared with other digested milk product controls**. All data was compared with the levels of positive control (H_2_O_2_/Menadione). All treatments were performed in triplicate and assay was repeated three times independently with similar results. Data are expressed as mean ± SD. **P < 0.001 is the highly significant value from the control with media only. *P < 0.05 is the significance value from the control with media only.

We examined the levels of AOE activities in Caco-2 cells treated with Enprocal D and compared with other control commercial milk product (WPC and P2) digests. There was no significant change in the activities of either CuZnSOD or MnSOD in Enprocal D and WPC treated cells as compared to vitamin C (positive control) treated cells (Fig. 6A). This indicates that while Enprocal D did not induce oxidative stress in the gut cells, P2 treatment led to highly significant (P < 0.001) increase in the activities of these enzymes indicating that it is inducing an oxidative stress response. Increased SOD activity if not accompanied by similar levels of other AOEs may be harmful. Activity levels for CAT were not significantly (P > 0.05) effected by the treatments with Enprocal, WPC and P2. However activity levels for GPx increased significantly (P < 0.01) after treatment with P2 digest (Fig 6B).

### Enprocal D upregulates the surface expression of co-stimulatory CD40, CD86, CD80 MHC I & MHC II on human macrophages and inhibits the monocyte/lymphocyte-epithelial cell adhesion

**Figure 7:**
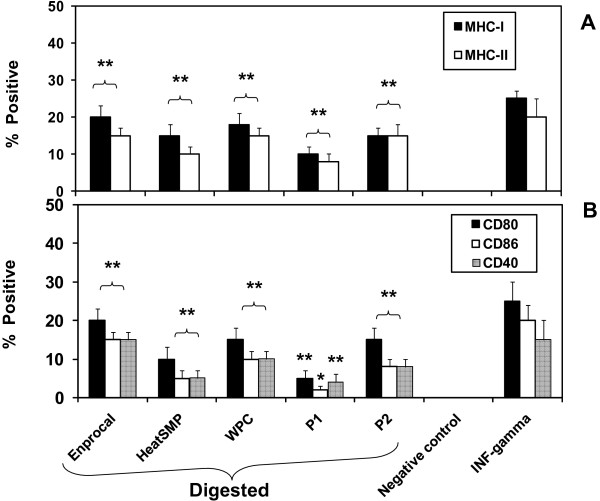
**Cell surface activation marker expression on the human THP-1 (PMA differentiated) macrophages for MHC-I and MHC-II molecules (*A*) and co-stimulatory (CD80, CD86 and CD40) molecules (*B*) is modulated by Enprocal D and compared with other digested milk product controls *in vitro***. Responses were measured by the upregulation of the surface markers CD80, CD86 and CD40. A negative control of cells without treatment is shown. Positive control of LPS and IFN-γ is shown. All treatments were performed in triplicate and assay was repeated three times independently with similar results. The mean for each experiment was calculated and presented as a mean ± SD values. ** Indicates a highly significant P < 0.001 value from the control with media only. *Indicates a significant P < 0.05 value from the control with media only.

In a transwell coculture model of Caco-2 and PMA differentiated THP-1 macrophages, movement of bioactive components of with Enprocal D and other digested milk product controls from mucosal (apical wells with Caco-2 monolayers) to serosal (basolateral wells containing PMA differentiated THP-1 macrophages) induced up-regulation of CD40, CD80, CD86 and MHC-I & II expression on THP-1 macrophages in a manner similar to that of immune stimulant IFN-γ (Fig 7A &7B). Although THP-1 macrophages showed no expression of activation markers in response to the negative control (no treatment; cells in culture medium alone), they strongly up-regulated expression of CD40 (P < 0.001), CD86 (P < 0.001), CD80 (P < 0.001), and MHC I & II (*P*< 0.001) on the cell surface upon treatment with Enprocal D, other digested milk product controls compared to no treatment control. The response observed was maximal with Enprocal D.

**Figure 8:**
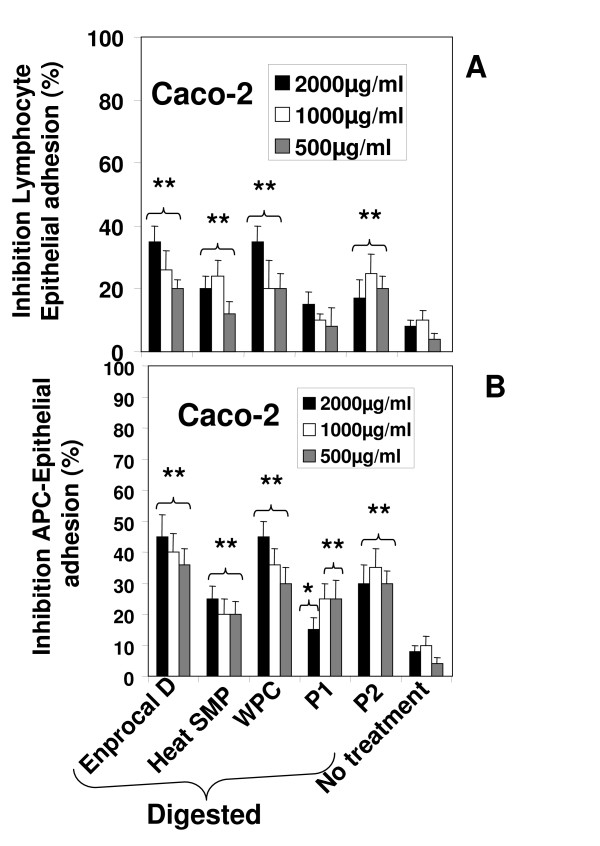
**Inhibition of lymphocyte-epithelial (A) and monocyte-epithelial (B) cell adhesion by Enprocal D and other digested milk product controls**. For adhesion assays, Caco-2 cell monolayers, grown in 96-well plates, were treated for 18 h with Enprocal D and other digested milk product controls. CMFDA-labeled Jurkat (A) or THP-1 (B) cells were then added. After incubation for 60 min at 37°C, nonadherent immune cells were removed by washing with HBSS and the monolayer-associated Jurkat cells or THP-1 was counted. Values are showed as the percent inhibition. All treatments were performed in triplicate and assay was repeated three times independently with similar results. The mean for representative experiment was calculated and presented as a mean ± SD values. ** Indicates a highly significant P < 0.001 value from the control with media only. *Indicates a significant P < 0.05 value from the control with media only.

Enprocal D in a dose dependant manner significantly (P < 0.001) inhibited the adhesion of lymphocytes and monocytes to Caco-2 cells. Similar levels of inhibition of adhesion were observed with P2 (Fig 8A &8B).

### Enprocal D modulates the cytokine response in human immune cells (down-regulates the proinflammatory cytokines TNF-a and Il-1β and up-regulates Il-2, IL-10 and IFN-γ)

The levels of proinflammatory (TNF-α and IL-1 β), both Th1 (IFN-γ and IL-2) and Th2 (IL-6 and IL-10) cytokines and chemokine IL-8 were measured in cell culture supernates of THP-1 (LPS stimulated) and Jurkat E6-1 cells. These immune cells were grown in lower (basolateral) wells of transwell coculture models of Caco-2 and THP-1 Caco-2 and Jurkat E6-1 cells. As shown in Table 3 the cytokine modulatory effect upon movement of bioactive components of Enprocal D and other digested milk product controls from mucosal (apical wells with Caco-2 monolayers) to serosal (lower basolateral wells) was evident. All the cytokine levels in the growth media samples and untreated cells were below the detection limits of ELISA. The cytokines response induced in the LPS only stimulated cells was highly significant (p < 0.001) as compared to non-stimulated cells and considered 100%. The levels of LPS stimulated TNF-α, IL-1β and IL-6 were reduced dramatically by 91%, 57% and 44% respectively after treating cells with Enprocal D as compared to LPS only control. WPC similarly reduced the levels by 85%, 75% and 84% for TNF-α, IL-1β and IL-6 respectively. However, with P2 control product levels of TNF-α and IL-6 were either negligible or below detection limits and it decreased the levels by 87% for IL-1 β. There was no effect on IL-8 production by any of the products tested as compared to maximal response. When compared with the positive control (PMA) Enprocal D treatment increased levels of IFN-γ by 29%, while both WPC and P2 decreased the levels of IFN-γ by 51% and 88% respectively in the supernates of Jurkat E6-1 cells. Enprocal D treatment increased levels of IL-10 by 48% compared with control PMA stimulated cells and WPC and P2 increased IL-10 by 26% and 15% respectively. Both Enprocal D and WPC induced IL-2 secretion in non-stimulated Jurkat cells to nearly the same (905 ± 12 pg/ml and 925 ± 12 pg/ml respectively) levels as were achieved with PMA stimulated cells (positive) control (955 ± 15 pg/ml). With P2 digest treatment the IL-2 secretion levels were decreased by 56% when compared with positive (PMA stimulated cells) control (100%).

**Table 3:** Effect of Enprocal D and other digested milk product controls on modulation of cytokine secretion determined by ELISA.

**Cells**	**Cytokines**	**Enprocal D**	**WPC**	**P2**	**Media +cells**	**Enzyme cocktail**	**LPS^a^/PMA^b^**
**THP-1 cells^a^**	**IL-1β**	305 ± 10**	180 ± 8**	120 ± 5**	UD	700 ± 32^c^	710 ± 35
	
	**TNF-α**	50 ± 2**	80 ± 2**	UD	UD	520 ± 27^c^	530 ± 28
	
	**IL-6**	70 ± 5**	20 ± 2	10 ± 2	UD	114 ± 4^c^	125 ± 4
	
	**IL-8**	1495 ± 15	1485 ± 12	1515 ± 16	UD	1500 ± 1^c^	1505 ± 15

**Jurkat cells^b^**	**IL-2**	905 ± 12	925 ± 10	535 ± 14**	UD	UD^d^	955 ± 15
	
	**IFN-γ**	105 ± 5	40 ± 5*	10 ± 2	UD	UD^d^	81 ± 5
	
	**IL-10**	135 ± 10**	115 ± 10*	105 ± 6*	UD	UD^d^	91 ± 6

UD = undetectable levels.

^a^Enprocal digested and other digested milk product controls were added to Caco-2 cells in apical (upper) wells and culture supernatants were collected from the THP-1 (LPS stimulated) cells grown in basolateral (bottom) chamber after 8 hours (TNF-α) and 24 hours (IL-1β, IL-6 and IL-8) incubation.

^b^Enprocal digested and other digested milk product controls were added to Caco-2 cells in apical wells and culture supernatants were collected from the Jurkat cells grown in basolateral chamber. Supernatant from Jurkat cells stimulated with PMA was used as positive control for the measurement of IL-2, INF-γ and IL-10 cytokines.

^c^Control THP-1 cells with enzyme cocktail and with LPS stimulation.

^d^Control Jurkat cells with enzyme cocktail and without PMA stimulation.

Concentration of cytokines in the culture medium was determined by ELISA as described in the Methods section. Cytokine concentrations in negative control (untreated cells) and growth media only were below the detection limits. All samples were assayed in triplicate and three independent experiments were performed. The mean for all experiment was calculated and presented as a mean ± SD values.

** Indicates a highly significant P < 0.001 value from the control with positive control (LPS/PMA) only. *Indicates a significant P < 0.05 value from the control with positive control (LPS/PMA) only.

## Discussion

In the present study, we reported for the first time the potential of Enprocal to improve gut and immune health. In the past decade, it has become clear that much of the gastrointestinal tract maintains youthful function well into the eighth and ninth decade of life. There is little difference between a 20 year-old and an 80 year-old in the quantity and quality of biliary, pancreatic and intestinal secretions, in the absorptive capacity of the small intestine, and general nutritional requirements [31]. In practical terms in gut health, swallowing and defecation are the two most likely functions to be affected by aging [31]. The areas at greatest risk of developing aging-related dysfunction are the upper GI tract, particularly the oropharynx and esophagus, and the distal tract (colon and rectum). The inability to eat sufficient amounts orally leads to an often compromised nutritional intake in the elderly. Their diets are low in protein, low in energy (more specifically energy density) and deficient in micronutrients [1,3,4] and elderly gut slows the absorption of nutrients from the gut lumen have also been reported [1,3]. Enprocal is a formulated supplement developed to meet the nutritional profile of elderly and contains whey protein concentrate, skim milk powder, whole milk powder, vitamins and minerals (including calcium, zinc and vitamins C, D, B group and A), vegetable oils and inulin (fibre).

### Enprocal D helps in gut cell growth, maintains gut barrier function and mucosal-integrity

Although not mimicking the ageing gut epithelium, FHs 74 Int and Caco-2 cell lines are employed world wide as suitable cell culture models displaying enterocyte like differentiation for studying the proliferation and gut integrity/permeability and immunomodulation at cellular level [23-30,32,33]. We first determined a concentration range of Enprocal, which was not cytotoxic (kill/disrupt) to the Caco-2 cell monolayers, needed to test the downstream intestinal permeability assays, adhesion assays etc. We used initially both undigested versus digested Enprocal to determine if there was any difference between the effects of two upon treatment with gut cocktail mixture on cell viability. Both digested and undigested Enprocal had no affect on Caco-2 cell viability over a dose range of 500–2000 μg/ml and this concentration range was chosen for bioassays. Since Enprocal is a dietary supplement to be taken orally and to mimic conditions as close as possible to those of the human digestive tract following oral route, we conducted all the assays with digested samples only. Other digested milk product controls were also tested for their effects over this dose range on Caco-2 cell viability and cell death (LDH release). At 500–2000 μg/ml, Caco-2 cells appeared to be resistant to the cell death/cytotoxic effects (apoptosis/necrotic) by Enprocal D after 48 hours as compared to other digested milk product controls. In particular Heat SMP has significantly increased the apoptosis/necrotic index indicating the presence of unidentified potential anti-cancer bioactive peptides released upon its digestion [11,12]. At higher doses (4000 and 8000 μg/ml) Enprocal (undigested and digested) induced cell death and loss of viability in Caco-2 cells but not in normal FHs 74 Int cells. Enprocal samples were found to provide normal intestinal epithelial cell protection as no cytotoxicity resulted towards FHs 74 Int cells after treatment with Enprocal (undigested and digested) at 100–8000 μg/ml tested in the LDH release assay. It has been reported that an α-lactoalbumin complex from acid-precipitated human milk casein (MAL) induces apoptosis in tumor cells (Caco-2 and HT-29) and immature cells, but not in mature differentiated FHs 74 Int cells [34,35]. In MTT cell proliferation assay, Enprocal D in a dose dependent manner stimulated the selective proliferation of normal FHs 74 Int cells and this stimulation was significantly (P < 0.001) more effective than positive control EGF. Due to an absence of cytotoxicity effects against these cells our results suggest that Enprocal is safe to be taken orally and may prove beneficial to premature infant gut growth. Further studies are needed to determine the mechanisms involved in growth-promoting effect of Enprocal on fetal small intestinal cells. Also the anti-cancer property of Enprocal in Caco-2 cells at higher doses is a matter of an interesting future investigation. The anti-cancer effect of Enprocal may be attributed to the activity of its WPC' components such as Lactoferrin, which have been shown to inhibit tumour cell growth both in vitro and in vivo [8,12,15,36] or the synergistic effect of WPC components with vitamins and calcium [37].

We further demonstrated that Enprocal improved gut cell wall integrity, aided in macromolecular absorption. Disruption of the epithelial barrier in the gut is a hallmark of gut inflammation and diseases. Failure of barrier function in the gut epithelium is a key feature in the pathology of diseases, such as leaky gut syndrome, IBD, cancer etc. [38]. Healthy intestinal tract acts a barrier against colonization and/or translocation of pathogens and toxic compounds. Diarrhoeal diseases as a result of gut infection caused by Escherichia coli, Salmonella, Shigella, Campylobacter jejuni, Entamoeba histolytica and rotavirus are one of the important causes of infant mortality and morbidity in developed and underdeveloped countries [39-41]. To ensure monolayer integrity, TEER of Caco-2 polarised monolayers grown on millicell cell culture inserts (Transwell system) was measured using Millicell-ERS system voltohm meter which measured the monolayer resistance under aseptic conditions. Compared with other control products, high TEER values were observed in monolayers' treated with Enprocal D and P2 after 6 hours of treatment. These values returned to baseline after 12 hours and after 24 hours TEER values went further up with only Enprocal D treatment and which were maintained till 48 hours. These observations indicate that there was no difference between Enprocal D and P2 with regards to the functional activities to gut cells following treatment at till 24 hours. The gut cell integrity results were again confirmed by expression of tight junction protein Zona Occludin (ZO-2) on treated Caco-2 monolayers. Enprocal D and P2 as compared to control products increased the expression of tight junction protein -ZO-2.

In the gut permeability assay, Enprocal D aided macromolecular transport studied by HRP-flux through the Caco-2 monolayer. The transport of nutrients across the intestinal epithelium may occur by one or more of 4 different routes: the passive transcellular and paracellular routes, the carrier mediated route or by transcytosis. Caco-2 monolayers have been world widely used to study drug transport by all 4 pathways [26,27] and permeability through Caco-2 cell monolayers correlates well with human in vivo absorption [27]. The results of the HRP flux indicated that HRP flow through increases with time after treatment with Enprocal D and other digested control milk products. This was reflected in the increased flux of HRP across the monolayers by the 4 hour point. All these results of TEER, HRP-flux and ZO-2 expression clearly indicates that Enprocal maintains the gut cell integrity even after digestion and showed no side effect on its absorption and permeability through Caco-2 monolayer.

### Enprocal treatment does not induce oxidative stress/injury

Increases in the levels of reactive oxygen species (ROS) that may occur during periods of oxidative stress, appear to be detected by redox-sensitive regulatory molecules in the cell, triggering homeostatic responses to prevent cellular injury [21]. Among those responses is the regulation of AOE, as the levels and balance of AOE modulate the susceptibility of the cell to oxidative injury. Highly reactive free radicals formed by exogenous chemicals or endogenous metabolic processes in the human body or in food systems are capable of oxidizing biomolecules, resulting in cell death and tissue damage. In the gut, variations in the activities of AOE have been reported under different inflammatory/pathologic conditions associated with free radical injury [31]. We examined the specific AOEs such as CuZnSOD, MnSOD, GPx, and CAT activities in Caco-2 epithelial cell cultures treated with Enprocal D and other digested milk product controls. With Enprocal D there were no significant changes in the induction of AOE activity levels and were similar in effect to natural anti-oxidant vitamin C used as a positive control. These results indicated that no oxidative stress or free radical injury occurred to the cells and these findings can be explained on the basis of synergistic effects of Enprocal product formulated with vitamin C, A, and Zn, the known anti-oxidants.

#### Enprocal up-regulates the surface expression of co-stimulatory (CD40, CD80 and CD86) molecules and MHC I and II on human macrophages

Macrophages as antigen-presenting cells (APCs) play an important role in innate immune response in the gut and represent one of the first lines of nonspecific defence against bacterial invasion. The macrophages are highly responsive to their environment (biosensors) and relay messages instructing other arms of the immune system e.g., T cells and B cells, to respond in an appropriate way [42]. These messages can be through costimulatory molecules (present on the cell surfaces that facilitate direct interactions between APCs and T cells) or soluble proteins (cytokines). Even though cytokines play diverse roles in regulating immune functions, some cytokines, e.g., tumor necrosis factor (TNF)-α, interleukin (IL)-1β and IL-6 have received more attention than others because they have traditionally been classified as proinflammatory and as such are known to be associated with chronic inflammation including IBD [5].

**Figure 9:**
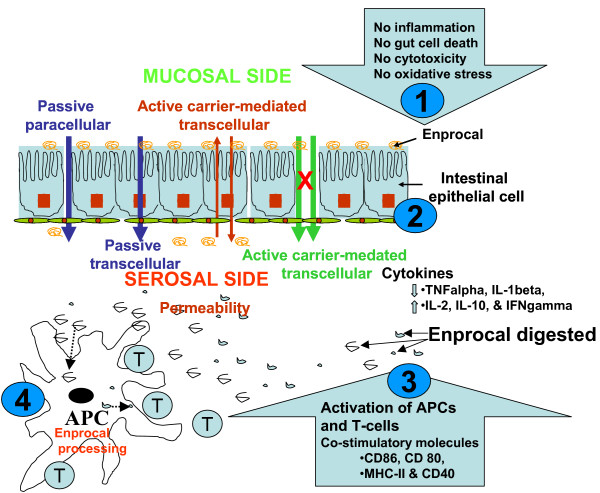
**Schematic drawing of a model on hypothesis of mechanism(s) of Enprocal's function in gut and immune health**. Enprocal D working model depicting the activation of immune and strong gut cell integrity. 1 & 2) Oral administration and its digestion of Enprocal D maintains the human gut cell integrity and aids in gut cell proliferation with no cytotoxicity and no oxidative injury. 3) Modulation of Th1 and Th2 cytokine responses by Enprocal D appear to aid the production of antibodies and gut immune functions. 4) By enhanced activation of immune responses, Enprocal thus, may prevent pathogen invasion. With the strong gut cell integrity and downregulation of proinflammatory cytokines such as TNFα and IL-1β, Enprocal D may help in the management of chronic gut inflammatory diseases such as inflammatory bowel disease (IBD) and leaky gut syndrome.

Recently an in vitro coculture system has been reported that could be utilized for an assay to search for drugs or food substances, which could prevent intestinal inflammation [33]. To study the cell-to-cell interaction between intestinal epithelial cells (Caco-2) and activated macrophages (THP-1), we utilized the similar Transwell model. We used THP-1 cell line rather than primary human monocytes or dendritic cells (DCs) to minimize the variability due to sample collection from different subjects. DCs are considered to be highly sensitive and professional APCs of the immune system [43], but they are difficult to isolate and require careful handling to prevent them becoming activated before treatment with any bioactive. Thus, they can produce variable results [42]. We found that digested Enprocal when added to apical (upper) wells containing Caco-2, up-regulated the expression of "activation markers" (CD 40, CD80, and CD86 and MHC I & MHC II) in PMA differentiated THP-1 macrophages cultured in bottom (basolateral) wells. This implies that as proposed in the hypothesis (working model) in Figure 9, Enprocal components moved from the 'mucosal' to 'serosal' side by any of the mechanisms shown and interact with 'APC' to induce upregulation of costimulatory molecules and aid gut-immune cell cross-talk.

The intestinal anti-inflammatory activity exerted by Enprocal was characterized by a down-regulation of cell adhesion of THP-1 and Jurkat E6-1 cells to Caco-2 monolayers treated with digested samples of Enprocal and other digested milk product controls in a static assay system. Although these adhesion assays do not replicate the in vivo conditions of dynamic gastrointestinal tract but these heterotypic assays have been used earlier for the development of immunomodulatory peptides [40]. Moreover it has been reported that there are peripheral (systemic) target sites for the biologically active milk-derived peptides that may be absorbed intact [reviewed in [11,12,44]] and enter the circulation. In that situation this anti-inflammatory activity of Enprocal may hold promise and clearly more work needs to be done in adhesion assays using normal primary human immune and epithelial/endothelial cells.

The intestinal intraepithelial lymphocytes and monocytes are effector cells capable of secreting cytokines in response to stimulation [45]. These cytokines include tumor necrosis factor-α (TNF-α), interferon-γ (IFN-γ) and interleukin (IL)-1β, IL-2, IL-6 and IL-10, and it has been suggested that IFN-γ and TNF-α can act directly on intestinal epithelial cells to mediate changes in the epithelial permeability and the capacity for electrogenic ion transport [46,47]. The mucosal to serosal movement of Enprocal D components' in our coculture system led to modulation of the cytokine secretion by monocytes and T-lymphocytes. In Enprocal D-treated THP-1 cell supernates, despite the levels of TNF-α and IL-1β being reduced, IL-6 (other proinflammatory molecule) secretion was not reduced to the scale of WPC. IL-2 (well known for its stimulatory role in T cell proliferation) secretion levels by Jurkat E6-1 cells were significantly higher with Enprocal treatment. Both IL-6 and IL-2 are important in the terminal differentiation of B-cells into plasma cells [48]. Enprocal D significantly enhanced the secretion of IFN-γ, and IL-10 compared to the positive control PMA, WPC and P2 treated T-lymphocytes. IL-10 is considered anti-inflammatory which inhibits the generation of cell mediated immune responses. IL-10 is an interesting multifunctional cytokine produced by T-cells, monocytes, and other cell types [49]. IL-10 enhances B-cell viability and augments the proliferation and differentiation of B-cells after stimulation through the Ig receptor [49,50], therefore associated with humoral or Type 2 immune responses. Overall, Enprocal D provided the most consistent up-regulation of anti inflammatory cytokines and was the second most prolific down-regulator of pro inflammatory cytokines. Enprocal, through down-regulation of proinflammatory cytokines such as TNF-α and IL-1β (both known to play an important role in gut chronic inflammation), may prove beneficial in IBD, CD & UC. TNF-α, in particular has been recognized as playing a central role in the pathogenesis of Crohn disease. Nevertheless, reducing the production or effect of TNF-α in Crohn disease patients is believed to be beneficial [51]. In fact, different drugs capable of interfering with the activity of TNF-α cytokine have been successfully developed for IBD therapy [52,53]. However, despite earlier hopes, the results from studies using TNF-α antagonists were disappointing, and there were some reports of severe complications [54].

## Conclusion

In conclusion, the schematic diagram shown in Figure 9 proposes a model (hypothesis) on mechanism(s) of Enprocal's function in gut and immune health. Following oral administration and upon digestion of Enprocal, amino acids and potentially bioactive di and tri-peptides, which may be released through enzymatic action from its key ingredients such as whey protein concentrate, aid in normal human intestinal cell proliferation with no cytotoxicity, maintain gut integrity, increase tight junction protein expression, and enhance activation of immune responses. Through modulation of the Th1 and Th2 cytokines responses Enprocal may aid the production of antibodies and gut immune functions. These observed effects of Enprocal however may arise not only from the presence of dairy proteins but also from synergistic relationships with its other components in particular the micro-nutrients. Strong gut cell integrity and activation of immune cells with Enprocal may prevent infections caused by pathogens or the invasion or entry of allergens. Thus in future the detailed studies should be carried out on efficacy of Enprocal in reducing individual pathogens and gut cell interactions.

Stimulation of fetal intestinal cell proliferation without cell cytotoxicity by Enprocal indicates that it may also be given as infant food particularly for premature babies. Although gut epithelial stem cells show no intrinsic limit to their proliferative capacity but recent evidence [reviewed in [55]] indicates that they suffer important functional impairments associated with an altered pattern of cellular response to DNA damage during the course of ageing. In future studies, it would also be worthwhile to determine whether consumption of Enprocal is effective in preventing such functional damage to the stem cells in the aged gut. Finally, our in vitro findings by employing well characterized currently available and widely used cell models may be relevant for gut and immune health of consumer groups with special needs, such as infants and the elderly. However future studies are needed to reveal the potential of Enprocal as a formulated supplement that produces defined effects on the human gut and immune health.

## Methods

### Enprocal and other commercially available milk products

Enprocal and other commercially available control milk products coded (P1 and P2) investigated in the study were obtained from Warrnambool Cheese and Butter Factory Company Holdings Limited (Victoria, Australia). The Composition of Enprocal is given in Table 1. Heat-treated skim milk powder (heat SMP) and whey protein concentrate 80 (WPC) were included as control milk products. We cannot reveal the identity of other milk products P1 and P2 due to commercial reasons. Protein concentrations were measured by Coomassie Plus-The Better Bradford Assay kit (Endogen). Products were assayed for lipopolysaccharide (LPS) contamination and the concentration of endotoxin was < 0.13 μg/kg.

### In vitro digestion

Enprocal and the control milk products were digested with digestive enzymes cocktail mixture using a modified protocol [56]. The enzyme concentrations in the commercially available enzyme tablet are shown in Table 4. After gastric phase digestion the solutions of protein fractions and enzyme tablet were made for small intestinal phase in 0.1 M NaHCO_3 _solution pH 7.2. The reaction mixture was set in 1:50 (enzyme: protein) ratio (standardized after preliminary observations) and incubated at 37°C in a shaker (250 rpm). Enzyme digests were collected after 1, 2, 4 and 6 hours incubation. The reaction was stopped by inactivating the enzymes at 42°C and aliquots were stored at -20°C till further use in cell culture studies. Digests were analyzed by SDS-PAGE (reducing & denaturing) electrophoresis (Figure 1).

**Table 4:** Digestive enzyme cocktail ingredients.

Pancreatin (4×)	1250 mg
Papain	150 mg

Bromelain	150 mg

Trypsin	125 mg

α-Chymotrypsin	3 mg

Lipase	50 mg

Amylase	50 mg

Rutin	100 mg

### Intestinal epithelial cell culture

Caco-2 (human colon adenocarcinoma), and FHs 74 Int (an adherent human primary fetal small intestinal) cell lines were obtained from American Type Culture Collection (ATCC, Rockville) and cultured in their respective media from ATCC as per its guidlines. Caco-2 cells were cultured routinely in Eagle's Minimum Essential Medium (EMEM) purchased from ATCC supplemented with 20% (v/v) heat-inactivated fetal calf serum (Sigma). FHs 74 Int cells were routinely grown in Hybri-Care Medium (ATCC: 46-X) supplemented with 30 ng/ml human recombinant epidermal growth factor (EGF; Affinity Bioreagents) with 10% fetal bovine serum. All cell lines were grown at 37°C, in an atmosphere of 5% CO_2 _and 95% relative humidity.

### Cell Viability assays

Caco-2 and FHs 74 Int cells were incubated with various concentrations of Enprocal (undigested) & digested (Enprocal D) and other digested milk product controls for 48 hours. Cells were trypsinized to obtain a cell suspension and all adherent and floating cells were collected for counting the live and dead cells under microscope following trypan blue staining.

### Lactate dehydrogenase (LDH) cytotoxicity assay

The cytotoxic effect of different test treatments on Caco-2 and FHs 74 Int was studied by a colorimetric LDH release assay using a commercially available kit (Roche Applied Science). This assay quantifies cell death and lysis based on measuring the release of lactate dehydrogenase (LDH) which is present within the supernatant following the loss of membrane integrity. The assay was carried out as per kit's instructions. Briefly, 2 × 10^3 ^cells per well (total volume 100 μL) were plated into a 96 well plate and incubated for 24 hours at 37°C in 5% CO_2_. Following incubation, growth media was replaced with fresh media containing different concentrations of Enprocal (undigested), Enprocal D (digested) and other digested milk product controls, and cells incubated further for 48 hours. Cytotoxicity measurements were tested at 48 hours after treatment with Enprocal and other digested milk product controls. Triton-X100 (1%V/V) was used as a high (positive) control and cells in media only was the low (negative) control. The amounts of lactate dehydrogenase (LDH) in the supernatant were determined and calculated as per kit instructions. All tests were performed in triplicate and assay was repeated three times independently with similar results.

### Apoptosis versus necrosis

In order to detect the nature of cell death following treatment with different test samples as well as to differentiate between apoptosis and necrosis at single cell level, Caco- 2 and FHs 74 Int cells were plated at a density of 1 × 10^4 ^cells on 8 well slides and grown for two days at 37°C with 5% CO_2_. Following media removal, the cells were then treated with different concentrations of Enprocal D and other digested milk product controls (in triplicates) in serum free media for 48 hours. As a positive control, apoptosis inducing anti-cancer drug Paclitaxel (BioVision), was added to the cells (final concentration of 20 nm). After 48 hours the media was removed and apoptotic cells were determined by TUNEL staining using an in situ apoptosis detection kit from Boehringer Mannheim as per kit instructions [57]. Slides were also stained with propidium iodide (Sigma-Aldrich) to distinguish necrotic cells from those undergoing apoptosis. Same slides were counterstained with methylene blue-staining and mounted. The total number of apoptotic or necrotic cells was counted. The apoptotic and the necrotic indices were calculated as follows: Apoptotic index (A/I) or necrotic index (N/I) = number of apoptotic or necrotic cells × 100/total number of nucleated cells. Following treatments, another kit, Annexin-VFLUOS staining (Roche Applied Science), was also used to differentiate apoptotic cells from necrotic cells, as per manufacturer's instructions. This assay is based on principle that phosphatidyl serine, located in the inner leaflet of the cell membrane, is exposed at the cell surface in the early stage of apoptosis. Annexin V shows high-affinity for phosphatidyl serine-binding that makes it a useful selective and powerful tool for detection of apoptotic cells [58].

### Cell proliferation (MTT) assay

Human intestinal cell lines Caco-2 and FHs 74 Int were seeded each into 96 well plates, at a density of 2 × 10^3 ^cells per well (total volume 100 μL) and incubated for 24 hours at 37°C in 5% CO2. After removal of media, the cells were incubated with fresh growth media containing different concentrations of Enprocal and other digested milk product controls, for 48 hours. As a positive control EGF 50 ng/ml was used. The effect of different treatments on cell proliferation of Caco-2 and FHs 74 Int cells was compared in treated versus untreated cells with the use of commercially available 3-(4,5-dimethylthiazol-2-yl)-2,5-diphenyltetrazolium bromide (MTT) assay Kit (R&D systems). The assay was carried out as per manufacturer's instructions. All tests were performed in triplicate and assay was repeated three times independently with similar results.

### Measurement of transepithelial resistance (TEER)

Mucosal integrity of Caco-2 cells seeded onto Milicell cell culture inserts (Millipore Australia) in 24-well plates was studied by measuring the transepithelial electrical resistance (TEER) with a Millicell-ERS voltohm meter (Millipore Australia). The device contained a pair of chopstick electrodes (which facilitated the measurements) and measures membrane potential and resistance of epithelial cells in culture. It does this by qualitatively measuring cell monolayer health and cell confluency [59]. Caco-2 cells were seeded at a density of 4 × 10^3 ^per well in the volume of 200 μl growth medium (EMEM plus 20% fetal bovine serum) added to the apical well of insert and 800 μl of growth medium was added to the basolateral well in 24-well tissue culture plates for incubation. To obtain monolayer, cells were grown for 21 days and medium was replaced every third day. After 21 days the monolayers were thoroughly checked for any gaps under the microscope in order to prevent any false TEER readings. The differentiation was characterized by a polarization of the monolayer with formation of domes. These filter-grown monolayers were first equilibrated with serum free growth medium (EMEM containing GIBCO's antibiotic-antimycotic supplement for 2 hours under cell culture conditions. TEER was measured at the start of experiment i.e. before the addition of Enprocal D and other digested milk product controls. Each preparation was added to the apical compartment in serum free growth medium, and Triton-X, was used as a positive control. All test and control samples were added in triplicate. Cell monolayers were incubated at 37°C, 5% CO_2 _and 90% humidity and TEER was measured at specific intervals (6, 12, 18 and 24 hours) and at the end of experiments (48 hour). The monolayers were incubated at 37°C in 5% CO_2 _and 90% humidity between measurements.

### Horseradish peroxidase (HRP) flux

Caco-2 cells were seeded on to Millicell cell culture inserts at a density of 4 × 10^3 ^per well and grown as monolayers for up to 21 days as described above. Following medium removal, the cells were equilibrated for 2 hours with Hanks Balanced Salt Solution (HBSS) without phenol red (Invitrogen) and then treated with Enprocal D and other digested milk product controls. Horseradish peroxidase (HRP-Sigma-Aldrich) was added to the apical side of the monolayer at a final concentration of 0.15 mg/ml [60]. Following incubation at 37°C in 5% CO_2 _and 90% humidity, the flux of HRP was measured at 4, 8 and16 hours incubation, by taking 25 μl from the apical compartment and 300 μl from the basolateral compartment. The HRP activities were then measured in aliquots of these samples by treating the samples with 3,3',5,5'-tetramethylbenzidine (TMB; Sigma-Aldrich) which is an HRP substrate and then measuring the absorbance of the samples using an ASYS Expert plus UV ELISA plate reader at 450 nm. All treatments were performed in triplicate and assay was repeated three times independently with similar results.

### Immunofluorescence staining for ZO-2 tight junction protein

Caco-2 cells were grown on sterile 8 chamber slides (BD Sciences) with seeding density of 1 × 10^4 ^cells/ml. After 5 days of growth monolayers, were equilibrated with serum free medium and then treated with Enprocal D and other digested milk product controls as well as 1% Triton-X (positive control) for 12 hours. Monolayers were washed with PBS (pH 7.2), fixed in 4% paraformaldehyde (in PBS; pH 7.2) for 15 minutes at room temperature (RT) and blocked for 30 minutes in 2% normal rabbit serum (Vector labs) in PBS. After 3 PBS washes monolayers were incubated with mouse monoclonal anti-ZO-2 (Invitrogen diluted 1:1000 in PBS containing 1% BSA) overnight at 4°C. Secondary anti-mouse IgG-FITC antibody (diluted 1:100 in PBS; Sigma-Aldrich) was used to detect the primary antibody staining (1 hour incubation at RT). After 3 PBS washes, chambers were removed from the slide and mounted with one drop of Vectashield mounting medium with DAPI (Vector Labs). For primary and secondary antibody controls, cells were stained as above except incubation with PBS containing 1% BSA was carried out at appropriate staining steps. The slides were then viewed with Leica TCS SP5 confocal system fitted with inverted DMI 6000 microscope. Images were collected with LAS AF software. These images were then processed in Adobe Photoshop CS3.

### Antioxidant enzyme (AOE activity assays

For each assay Caco-2, 2 × 10^4 ^cells/well were grown in 6 well plates for 24 hours and then treated with Enprocal D and other digested milk product controls for 48 hours. Following incubation, cells were isolated in assay buffer and lysed by a freeze-thaw cycle. The debris was removed by centrifugation at 8000 × *g *for 5 min at 4°C, and the protein content of each sample was determined using the Coomassie Plus – The Better Bradford™ Assay Kit (Endogen). AOE activity assays were carried out spectrophotometrically by the methods as described earlier for superoxide dismutase (SOD) [61], glutathione peroxidase (GPx) [62] and catalase (CAT) [63]. All treatments were performed in triplicate and assays were repeated three times independently with similar results.

#### Superoxide dismutase (SOD) activity assay

To the assay mixture containing 0.1-mM cytochrome *c*, 0.05-mM xanthine, and KCN (10 μM for total SOD activity, 3 mM for MnSOD activity) in phosphate buffer, 150-μg protein (for each sample) was added in a final volume of 3 ml. After zeroing (blanking), xanthine oxidase was added to a final concentration of 0.6 U/ml, and the change in absorbance at OD at 550 nm was recorded at regular intervals over 4 min. A standard curve for SOD was generated and enzyme activity was determined by comparing samples to a standard curve of known SOD activity. Cu,Zn-superoxide dismutase (CuZnSOD), activity was calculated as the difference between total SOD activity and Mn-superoxide dismutase (MnSOD) activity [61].

#### Glutathione peroxidase (GPx) activity assay

For GPx enzyme activity measurements aliquots of protein samples (140 μg of protein) were added to the assay mixture containing 1 U/ml glutathione reductase and 2 mM glutathione in 1 ml of phosphate buffer after 30 min incubated at 37°C for 30 min. Nicotinamide adenine dinucleotide phosphate and tert-butylhydroperoxide were added to final concentrations of 580 μM, respectively, and the change in absorbance at OD at 340 nm was recorded at regular intervals over 4 min. The amount of GPx required to oxidize 1 μmol nicotinamide adenine dinucleotide phosphate in 1 min at 25°C was defined as enzyme units and calculated on the basis of a molar absorptivity for nicotinamide adenine dinucleotide phosphate at 340 nm of 6.22 × 10^-6 ^[62].

#### Catalase (CAT) activity assay

CAT enzyme activity was measured by monitoring the decrease in H_2_O_2 _concentration spectrophotometrically over time. For each sample, 130 μg protein was added to 50-mM phosphate buffer in a quartz cuvette and, after blanking, H_2_O_2 _was added to a final concentration of 10 mM in 0.9 ml, and the absorbance at OD at 240 nm was recorded at regular intervals over 4 min. The specific activity of each sample was calculated based on activity of pure CAT as described earlier [63].

### Immune cell culture

THP-1 and Jurkat clone E6-1 obtained from American Type Culture Collection (ATCC Rockville, MD) were employed in immune assays. THP-1 cells are human myelomonocytic cell line that is widely used to study monocyte/macrophage biology in cell culture systems and Jurkat cells are human T lymphocyte (T cell leukemia). THP-1 cells were cultured and maintained in RPMI 1640 (Gibco-BRL), supplemented with 10% heat-inactivated fetal calf serum, 2 mM L-glutamine, 0.05 mM 2-mercaptoethanol,100 U/ml penicillin, and 100 μg/ml streptomycin (complete RPMI 1640). Jurkat E6-1 cells were routinely grown in suspension in RPMI-1640 medium supplemented with 10% heat-inactivated fetal bovine serum (FBS), 2 mM L-glutamine, 100 U/ml penicillin, and 100 μg/ml streptomycin.

### Monocyte/lymphocyte-epithelial adhesion assay

Adhesion assays were performed with THP-1 and Jurkat E6-1 cells under static conditions by the methods as described earlier [32,64] with modifications. For static adhesion assays Caco-2 cells were seeded at a density of 2 × 10^4 ^cells per well (total volume 100 μL) and grown as confluent monolayers in 96-well plates and preactivated with IL-1β 10 ng/ml overnight before adhesion assay. Washed monolayers were then treated for 18 h with Enprocal D and other digested milk product controls. Epithelial cells were washed twice with culture medium before addition of 100 μl (1 × 10^5^) labeled THP-1 or Jurkat E6-1 cells per well. Both THP-1 and Jurkat E6-1 cells were labeled with the fluorescent dye chloromethyl fluorescein diacetate (CMFDA; Molecular Probes, Oregon) at 37°C for 30 min [64]. The plates were incubated for 60 min at 37°C. After incubation, the monolayer was gently washed three times with PBS to remove non adherent cells. Fluorescence-labeled adherent cells were counted under fluorescent microscope in minimum of 5 fields.

### Cell surface marker staining

We employed a coculture model to examine the effect of Enprocal D treatment on the interaction between gut-immune cells. For the coculture experiments THP-1 monocytic cells were grown in 24-well plates at a density of 5 × 10^5 ^cells/well and differentiated into macrophage-like cells, by treatment with 200 nM phorbol myristrate acetate (PMA; Sigma-Aldrich) for 4 days [33]. The differentiated status was confirmed on the basis of morphology and staining for the expression of leukocyte integrin CD11b (macrophage-1 antigen) (data not shown). The semipermeable support membrane (Milicell cell culture inserts from Millipore) on which the intact Caco-2 cell monolayers had been cultured for 21 days was placed on the differentiated THP-1 cells that had been cultured on the 24-well plates. After starting the coculture for 18 hours, apical media was removed and Caco-2 cells were treated with fresh growth media containing Enprocal D and other digested milk product controls. Control treatments included cells in medium only (negative control), enzyme cocktail. Interferon-γ (IFN-γ) at a concentration of 5 ng/ml was added to the positive control wells on the basolateral compartment containing differentiated THP-1 cells. Cells were incubated at 37°C with 5% CO_2 _for 24 h and cell surface marker staining was determined by direct immunofluorescence in paraformaldehyde fixed (4% PF in PBS pH 7.2; 15 minutes at RT) cells. Cells were incubated for 1 hour at room temperature with FITC labeled antibodies to anti-human CD40, CD86, CD80, major histocompatibility complex (MHC) I & II (PharMingen and R&D Systems). Following washing with PBS buffer wells were mounted with one drop of Vectashield mounting medium with DAPI (Vector Labs). For primary and secondary antibody controls, cells were stained with PBS containing 1% BSA (no primary antibody). The presence of labeled cell surface markers was counted under the fluorescence microscope and percentage of positive cells was calculated from total number of cells (DAPI positive).

### Cytokine ELISA

In the Caco-2 THP-1 coculture model, cytokine levels were measured in the supernatant of LPS stimulated THP-1 cells grown in the lower chamber for 8 and 24 hours after the addition of Enprocal D and other digested milk product controls to Caco-2 cell monolayers in the upper chamber [33]. Briefly, THP-1 cells were grown and maintained in endotoxin free complete RPMI-1640 at the exponential phase. To increase the sensitivity to LPS, cells were stimulated with 5 ng/ml human recombinant IFN-γ (BioVision >98% with endotoxin level < 0.1 ng per μg of IFN-γ) for 16 hours [65,66]. Following incubation cells were centrifuged, re-resuspended in serum free medium and seeded in 24 well plates with final cell concentration of 8 × 10^5 ^cells per well. Caco-2 monolayers (21 days old) grown and differentiated on the Milicell cell culture inserts (Millipore) were placed on the wells containing THP-1 cells in 24-well plates. All cells were allowed to settle for 1 h and following removal of apical media, Enprocal D and other digested milk product controls were added to the Caco-2 cells in fresh growth media 30 minutes before LPS (50 ng/ml; E. coli, Sigma-Aldrich) was added to the THP-1 cells (lower basolateral chamber). Supernatants were collected after 8 (for TNF-a) and 24 hours for other cytokines at 37°C with 5% CO_2 _Supernants of Jurkat E6-1 cells grown in coculture with Caco-2 were used to determine the protein levels of IL-2, IL-10 and IFN-γ cytokines. Jurkat E6-1 cells were kept in the exponential growth phase by passages at 2–3 days intervals. As described above, Caco-2 monolayers (21 days old) grown and differentiated on the Milicell cell culture inserts (Millipore) were placed on the wells containing 2.5 × 10^5^cells/ml Jurkat E6-1 cells grown overnight. After one hour incubation, apical media was replaced with media containing Enprocal D and other digested milk product controls and incubated for 24 hours. PMA (20 ng/ml) was used a positive control. Jurkat cell supernatants were obtained via centrifugation at 4°C and stored at -80°C until cytokine analysis.

Amounts of the cytokines TNF-a, IL-1β, and IL-6 and IL-8, IL-2, IL-10 and IFN-γ in cell culture supernatants were determined by using Biotrak Easy ELISA kits (Amersham Biosciences) for human cytokines. The limits of detection of the assays were 2.5 pg/ml for TNF-a, 1.1 pg/ml for IL-1β, 1.4 pg/ml for IL-6, < 7.26 pg/ml for IL-8, 3.5 pg/ml for IL-2, 1 pg/ml for both IFN-γ and IL-10. Each assay protocol was carried out as per kit instructions using cytokine standards supplied with kit. Samples were run in triplicate and experiments were performed three times. The concentration of cytokines was determined by the standard curve using linear regression curve fit method.

### Data interpretation and statistical analysis

For the statistical evaluation of numerical data, one way ANOVA and Students t-test were used to compute the differences. In all cases, *P *< 0.05 and P < 0.001 were taken to identify levels of significance.

## Abbreviations

APC: antigen-presenting cell; AOE: Antioxidant enzyme; CD: cluster designation; CMI: cell-mediated immunity; DC: dendritic cell; Enprocal D: digested Enprocal; HRP: horseradish peroxidase; IL: interleukin; IFN-γ: interferon-γ; LPS: lipopolysaccharide; MHC: major histocompatibility complex; TGF-β: transforming growth factor-β; TNF-α: tumor necrosis factor-α; TEER: transepithelial resistance; SOD: superoxide dismutase; GPx: glutathione peroxidase; CAT: catalase; AI: apoptotic index; NI: necrotic index; LDH: lactate dehydrogenase

## Authors' contributions

JRK designed and carried-out immunomodulatory, cell adhesion and anti-oxidant experiments. RKK designed and performed the, cell integrity, permeability assays, confocal microscopy and cytokine measurements. JRK and RKK contributed equally to the manuscript. Authors read and approved the final manuscript for publication.
